# Influence of the quality implementation of a physical education curriculum on the physical development and physical fitness of children

**DOI:** 10.1186/1471-2458-12-61

**Published:** 2012-01-20

**Authors:** Gregor Starc, Janko Strel

**Affiliations:** 1 Faculty of Sport, University of Ljubljana, Gortanova 22, SI-1000 Ljubljana, Slovenia

## Abstract

**Background:**

This study was constructed as a comparison group pre-test/post-test quasi-experiment to assess the effect of the implementation of the PE curriculum by specialist PE teachers on children's physical development and physical fitness.

**Methods:**

146 classes from 66 Slovenian primary schools were assigned to quasi-test (71) and quasi-control (75) groups. Data from the SLOFIT database was used to compare the differences in physical fitness and development between groups of children whose PE lessons were delivered by specialist PE teachers from the second grade onwards (quasi-test, n = 950) or by generalist teachers in all first three grades (quasi-control, n = 994). The Linear Mixed Model was used to test the influence of specialist PE teachers' teaching.

**Results:**

The quasi-control group showed significantly lower improvement of physical fitness by -0.07 z-score units (95% CI -0.12 to 0.02) compared to the quasi-test group. A significant difference of -0.20 (-0.27 to -0.13) was observed in explosive strength, and of -0.15 (-0.23 to -0.08) in running speed, and in flexibility by -0.22 (-0.29 to -0.14). No significant differences in physical development were observed.

**Conclusions:**

Specialist PE teachers were more successful than generalist teachers in achieving greater improvement of children's physical fitness, but no differences were observed in physical development of quasi-test and quasi-control group.

## Background

Obesity, poor physical fitness of children and their causal dependency are associated with many preventable diseases and present a serious current and future public health problem [1]. Regular and quality physical activity during childhood is one part of the equation (quality nutrition being the other) that can lead to improvements in numerous physiological and morphological variables in children [2]. In addition, there are also numerous other benefits of physical activity on children's psychological development [3-5], lifestyle development [6-8], social development [9,10] and cognitive development [11-13]. A considerable part of children's physical activity is presently allocated to regular physical education (PE) classes in schools [14], because economic pressures [15] and parental concern for safety [16,17] often reduce children's physical activity in non-school settings. In Slovenia, this is especially problematic in the first years of school, when PE classes are frequently delivered by generalist teachers without appropriate PE teaching competences [18-25], because this can result in less effective physical fitness development, followed by increased risks of obesity and diminishing of motor skills and functional abilities, which then can lead to unfavourable results in adulthood [26].

The existing evidence suggests that activities for children have to be organised to be effective, because idle leisure, e.g. summer holidays, seem to be counterproductive for physical and motor development [27,28], and because there seem to be no difference between obese and non-obese children in unorganised leisure-time activities [29]. PE as an organised and compulsory activity could, therefore, be one of few possible environments for the successful intervention against the health-risk problems related to physical inactivity and obesity.

Often authorities try to improve negative trends with special interventions in schools but such programmes usually fail to produce considerable positive long-term effects [30]. The interventions usually include the allocation of additional time to physical education [31-33], specially designed after-school programmes [34,35], or a changed design of PE delivery [36]. However, this brings demands for additional temporal, spatial, human and economic resources.

In addition, the existing evidence shows that programmes with compulsory physical activity components, such as regular PE classes, seem to be superior to those based on educational interventions, as adherence is guaranteed [37]. Therefore, we tried to determine whether the negative trends can be improved within the educational system and existing PE curriculum, without time-limited and thus economically less effective external interventions or special voluntary intervention programmes, and with the partial allocation of already existing human resources in schools.

We believe that quality of PE depends on five factors: allocated time, available facilities and equipment, the contents of the PE curriculum, the number of children per teacher, and teacher competencies. Among these factors, we see that PE curriculum and its quality implementation are the determining factors of the PE outcomes. The official PE curriculums form the framework of possible interventions and in some cases can limit the effect of the subject. In the Slovenian case, the PE curriculum underemphasises health-related contents or considers them unintended collateral effects of the development of motor skills and the acquirement of sporting skills. The implementation of the curriculum, in contrast, depends on teachers' teaching competencies; our goal was to see whether specialist PE teachers' competencies have an effect on children's physical fitness and physical development by excluding the other four factors. During their 5 years of study, Slovenian generalist teachers receive between 175 and 355 h of PE teaching-related instruction, while the specialist PE teachers receive over 1,600 h; we attempted to verify whether this discrepancy in time allocated to the development of teaching competencies influences the quality and effectiveness of planning and teaching competencies of both profiles.

In Slovenia, specialist PE teachers are allowed to teach PE in the first 3 years of primary school, but this is currently considered a supplemental programme, requiring additional funding from parents or/and local communities, and the consent of the school board. If the school decides to have a specialist PE teacher teaching in the first 3 years, the classroom teacher has to be present in the class during these lessons. This gave us the opportunity to compare the physical fitness and physical development of the minority of Slovenian children whose PE classes in the first years of school are delivered by specialist PE teachers and of the majority of children who are taught only by generalist teachers.

## Methods

### Participants

**Figure 1:**
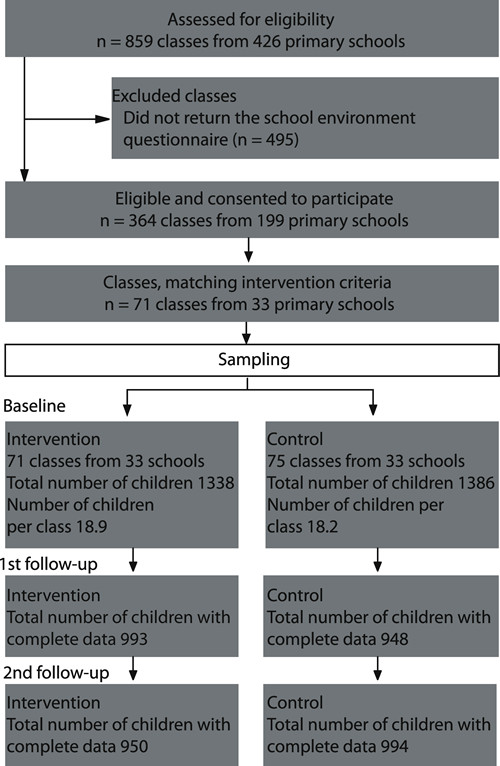
Flow of school classes and sample sizes throughout the quasi-experiment.

The sample consisted of 146 second-grade classes from 33 quasi-test and 33 quasi-control schools with 950 children in the first group and 994 children in the latter one (Figure 1). The sample included 453 girls and 497 boys in the quasi-test group and 483 girls and 511 boys in quasi-control one. The baseline age in both groups was almost identical (mean = 6.78, SD = 0.30).

### Procedures

In 2008, a questionnaire on school environment was sent to all 450 Slovenian primary schools and was completed by school principals or PE teachers. Among the 199 schools that returned the questionnaire, the majority had PE in the first three grades delivered by generalist teachers, while 33 had PE delivered by specialist PE teachers from the second grade onwards. The second-grade classes from the latter schools were assigned to the quasi-test group. We then paired the quasi-test classes with quasi-control classes from the neighbouring schools to exclude as many environmental factors as possible. PE and classroom teachers both followed the same official PE curriculum and had very similar teaching environments regarding facilities and equipment (all included primary schools have two gyms with standardised equipment), and number of children in the classes (see Figure 1); all classes had three compulsory 45-minute lessons of PE per week.

We used the SLOFIT database to extract data of the eight motor tests and the three anthropometric measurements for every child included in the first, second and third years of schooling. The results of the motor tests were used to calculate the physical fitness index (PFI) and the body mass index (BMI) for every child in all three grades. Baseline and both follow-up measurements took place at schools during PE lessons in April 2007, 2008 and 2009. The SLOFIT system, implemented in 1987 and formerly known as the Sports Educational Chart, is a Slovenian monitoring system of children's motor and physical development. Every April, qualified PE teachers with a completed anthropometry measurement course perform the measurements in all primary and secondary schools as required by the PE curriculum, following the official measurement protocol [38]. The SLOFIT test battery includes eight motor tests (arm-plate tapping (APT), standing long jump (SLJ), polygon backwards (PB), sit-ups (SU), standing reach touch (SRT), bent arm hang (BAH), 60-meter run (60 m) and 600-meter run (600 m)), and three anthropometric measurements (height (BH), weight (BW) and triceps skinfold thickness (TSF)). Measurements are always organised in school gyms between 08:00 and 14:00. Subjects were weighed barefoot in their shorts and T-shirts to the nearest 0.1 kg with portable scales of various brands; height was measured with stadiometers of various brands to the nearest 0.1 cm; triceps skinfold was measured with Holtain-Tanner callipers to the nearest mm. All instruments were calibrated once at the beginning of the measurements. Data were checked to detect coding errors. In order to include and evaluate the children's measurements in the SLOFIT system, and to use the data for scientific purposes, children need the written consent of their parents; throughout the existence of this system, the response rates in primary schools have remained above 94%. The SLOFIT database currently includes more than five million sets of measurements and grows at a rate of approximately 210,000 sets of measurements per year.

### Statistical analyses

All statistics were computed using the PASW 18 for Mac statistical software package (SPSS Inc., Chicago, IL). Since this was a quasi-experiment, we used the Linear Mixed Model procedure to test the influence of specialist PE teachers' teaching (PTE) on the physical fitness and physical development of children by excluding gender and age, and by using teacher and age in months as a fixed effect. Because there are individual differences among PE teachers, PTE was also used as a random effect along with schools. The physical fitness of children was assessed according to PFI, which was computed by averaging the z-scores of all eight motor tests. BMI was calculated from body weight and height, but the BMI z-score was used in the analysis. Linear Mixed Models were used to test for dependent variable PTE with independent variables. The latter consisted of two main primary outcome variables (PFI and BMI z-score), and secondary outcome variables (z-transformed results of individual motor tests in all three grades). Preliminary tests to identify possible effects of gender and school grade on PFI, BMI and secondary outcome variables were not performed, since z-scores of all tests were calculated by the RANKIT procedure according to school grade and gender. Several models and unstructured covariance matrix were tested separately for each dependent variable to find the best-fit model.

### Ethics approval

This study was funded by the Slovenian Ministry of Education and Sport and was approved by the Faculty of Sport of the University of Ljubljana ethics committee. Written positive consent of parents was provided for the use of data in scientific purposes. All procedures and methods in this study conformed to the ethics guidelines established by the World Medical Association's Declaration of Helsinki and the subsequent revisions.

## Results

Table 1 shows the results of the outcomes at baseline and both follow-ups, including adjusted differences at follow-ups.

**Table 1:** Measurements of anthropometric and motor tests at baseline and follow-ups

		Quasi-test			Quasi-control		Adjusted difference at follow-ups*	
**Variables**	**Baseline**	**Follow-up 1**	**Follow-up 2**	**Baseline**	**Follow-up 1**	**Follow-up 2**	**95% CI**	***P *value**

PFI (z-score)	0.02 (0.60)	0.06 (0.65)	0.08 (0.66)	-0.02 (0.60)	-0.04 (0.64)	-0.06 (0.66)	-0.07 (-0.12, 0.02)	0.006

BMI (kg/m2)**	16.24 (2.19)	16.94 (2.49)	17.60 (2.74)	16.05 (2.16)	16.90 (2.44)	17.53 (2.77)	-0.05 (-0.03, 0.13)	0.242

TSF (mm)**	10.53 (4.15)	11.73 (4.91)	12.43 (5.05)	10.63 (4.23)	11.64 (4.83)	12.24 (5.25)	0.02 (-0.06, 0.10)	0.624

BW (kg)	25.50 (4.74)	28.97 (5.77)	32.87 (6.84)	25.15 (4.57)	28.83 (5.59)	32.54 (6.75)	-0.03 (-0.12, 0.05)	0.451

BH (cm)	124.95 (5.32)	130.37 (5.51)	136.20 (5.87)	124.85 (5.23)	130.20 (5.28)	135.80 (5.63)	-0.04 (-0.13, 0.05)	0.368

APT (rep/20s)	22.61 (3.55)	25.94 (3.84)	29.01 (3.91)	22.46 (4.00)	25.92 (4.38)	28.98 (4.36)	-0.03 (-0.11, 0.04)	0.351

SU (rep/60s)	25.56 (7.62)	31.28 (7.78)	35.16 (8.61)	26.24 (7.65)	30.57 (7.54)	34.50 (7.79)	-0.02 (-0.09, 0.05)	0.619

SRT (cm)	43.55 (5.69)	44.03 (6.09)	44.55 (6.25)	42.59 (5.95)	43.07 (6.34)	42.69 (6.36)	-0.22 (-0.29, - 0.14)	<0.001

SLJ (cm)	120.69 (17.07)	133.34 (17.62)	142.69 (18.55)	117.97 (17.58)	128.60 (17.67)	138.08 (19.03)	-0.20 (-0.27, - 0.13)	<0.001

BAH (s)	21.80 (18.92)	27.99 (22.62)	30.84 (23.87)	21.51 (17.53)	26.09 (20.28)	30.42 (23.57)	-0.02 (-0.09, 0.06)	0.606

PB (s)**	22.51 (6.84)	17.82 (4.87)	16.08 (4.55)	22.04 (6.63)	18.02 (5.04)	16.38 (4.89)	-0.01 (-0.07, 0.07)	0.883

600m (s)**	206.56 (33.17)	194.08 (33.95)	181.23 (31.06)	207.69 (32.07)	191.47 (27.81)	182.81 (28.87)	-0.05 (-0.13, 0.03)	0.200

60m (s)**	13.10 (1.20)	12.28 (1.16)	11.67 (1.08)	13.26 (1.36)	12.47 (1.20)	11.88 (1.12)	-0.15 (-0.23, - 0.08)	<0.001

Values are raw (SD) unless stated otherwise.

*Adjusted difference in average z-score of respective outcome between quasi-test and quasi-control group with 95% confidence interval and P value, adjusted for gender and grade in Linear Mixed Model with random effect for PTE and school

**Z-scores ranked by lowest value. Higher z-score value signifies better result

In comparison to the quasi-test group, the quasi-control group showed significantly smaller improvement of physical fitness by -0.07 z-score units (95% confidence interval -0.12 to 0.02). In individual motor abilities, the quasi-control group significantly lagged behind the quasi-test group in relative explosive strength and running speed: in SLJ by -0.20 (-0.27 to -0.13) or 1.2% of mean baseline value and in 60 m by -0.15 (-0.23 to -0.08) z-score units or 0.5% of mean baseline value. The biggest difference occurred in relative flexibility (SRT), where the quasi-control group achieved poorer results by -0.22 z-score units (-0.29 to -0.14), representing an improvement of 2% of the mean baseline value of the quasi-test group. If observed through the percentage of change of baseline values to the second follow-up, the quasi-test group progressed 2.6 times more in PFI, 2.0 times more in SLJ, 1.8 times more in 60 m and even 4.1 times more in SRT than the quasi-control group. All other indicators of physical fitness from baseline to second follow-up showed a non-significant trend, but all in favour of the quasi-test group. Regarding physical development, no significant differences were observed between the quasi-test and the quasi-control group from baseline to the second follow-up.

## Discussion

With this quasi-experiment, we were able to show that the existing PE curriculum, planned and delivered by specialist PE teachers with higher PE teaching competencies than generalist teachers, positively affects children's physical fitness; however, it does not have such a distinct effect on their body composition. To our knowledge, no research has been conducted this far to investigate the effect of the quality implementation of the existing PE curricula on children's PFI and BMI, although there have been attempts to test the efficacy of PE programmes according to the exposure to PE [31].

Our findings are congruent with the findings of some intervention studies that have proven that school interventions can work if they are appropriately implemented and delivered by qualified professionals [39], and that PE specialists provide more effective physical education than non-specialists [40].

The quasi-experiment showed that the planning and delivery of PE lessons by PE specialist teachers in comparison to generalist teachers resulted in a relative improvement of physical fitness in the quasi-test group in comparison to the quasi-control group.

The less distinct influence on body composition could be connected to the existing Slovenian PE curriculum [41], which focuses mostly on motor development and especially on muscular fitness, but less on health-related issues like obesity. Research on the Slovenian PE teachers' competencies revealed that they feel very competent in the area of motor development [42], while evidence from other countries shows that, because of their low confidence in PE teaching competencies, many classroom teachers would prefer PE teachers teaching their PE classes [22]. An additional argument in favour of the higher quality planning and implementation of PE curriculum by specialist PE teachers is the observation that the quasi-test group improved their motor skills despite the growing burden of their subcutaneous fat.

The existing curriculum also encourages anaerobic activities over aerobic ones and the quasi-experiment confirmed that specialist PE teachers were more effective in improving anaerobic abilities than the generalist teachers, while no difference in aerobic abilities was observed. Since the quasi-test group improved their explosive strength and speed significantly more than the quasi-control group, it could be argued that the implementation of PE classes by specialist teachers can have a long-term positive effect on the enhancement of bone mass, which is related to muscular fitness in the paediatric population [43-46]. Quality PE from the beginning of schooling is important in this regard, because the evidence shows that participation in regular and intensive physical activity should start before the pubertal growth spurt to achieve the maximum development of bone as well as muscle mass [47,48].

Because there is evidence that flexibility does not normally increase with age [49], the four times greater increase of this motor ability in the quasi-test group could be attributed to environmental factors, including the higher quality of lesson delivery by specialist PE teachers compared to generalist teachers. Although there is not much evidence on the effects of flexibility on children's health, this component of neuromotor fitness seems to lower the risks of injury [50,51] and in this way contributes to children's health status.

The existing evidence proves that children in schools with fewer students per physical educator are more physically active during PE lessons [52]; this factor was not decisive in our case, since the average number of children per PE teacher was similar in both groups.

### Limitations

There are limitations to our study, and care should be taken in generalising its results to different countries, since there are considerable differences in the organisation and contents of PE curricula worldwide. The study was a quasi-experiment and did not control for many important environmental and social factors influencing the physical and motor development of children, although we tried to control for those factors by sampling from neighbouring schools from similar social and natural environments. The schools from the intervention groups were not randomly selected but were included based on the return of the school environment questionnaire. We were also unable to gather the information on teachers' actual PE planning and teaching competencies, which surely influence the quality of curriculum delivery. We were not able to gather the information on the intensity levels of PE lessons, which undoubtedly affect the health outcomes. Also, we gathered no information on the actual role of generalist teachers in PE planning and the delivery of PE lessons by PE specialists. In our experience, classroom teachers are present in the gym with the PE specialist but limit their activity to occasional help in setting the teaching environment and managing children who do not participate in the activities due to health and other reasons; they leave the planning and/or delivering the lessons to the PE specialists. We are aware that the level of physical fitness and body composition are not the only factors determining physical activity and consequent health outcomes in adult life, but the nature of our study did not allow us to gather additional information on the psycho-social effects of quality PE. Although the quasi-test and control groups had identical amounts of PE lessons per year, we have no information whether the children's out-of-school activities affected the results. Finally, we acknowledge that the quality of PE teaching does not rely solely on the level of professional competencies acquired in the teaching education programmes, but also on the level of social and other competencies of teachers that remained unknown to us.

## Conclusions

Our study shows that teachers' higher competencies in planning and delivering PE lessons positively contribute mostly to children's physical fitness and less to their body composition. The results suggest that specialist PE teachers seem to be more effective than generalist teachers in delivering of PE lessons, even if the learning environment, facilities and available equipment are very similar, if the curriculum is identical, and even with a similar number of children per teacher at PE lessons. Specialist PE teachers seem to deliver more effective PE lessons of seemingly higher intensity and have a consequently stronger positive effect on children's motor development, but not as significant an effect on their physical development. The contents of the curriculum are important in this regard, and we assume that a more balanced curriculum, including emphasis on health goals related to the decrease of children obesity, would have a stronger positive influence on the body composition of the quasi-test group. The main goal of PE should remain the enhancement of cardiovascular, motor and neuromotor fitness through vigorous physical activity, but some more emphasis should be put also on the promotion of positive health behaviours. Care must be taken not to base unrealistic aims for public health on school PE since teachers' primary task is teaching and not physical training or disease prevention; however, PE should not be ignored for its possible positive synergetic effects in the wider public health struggle against obesity and related health risks. To achieve more significant positive effects on body composition and consequent health, three 45-minute classes of PE per week seem to be insufficient, and we would suggest having five classes of at least 45 min per week with at least 60% of time spent on the level of moderate-to-vigorous physical activity. The evidence from other countries shows that the intensity of PE lessons is below recommended values, with Dutch children achieving 46.7% [53] and some US children merely 8.6% [54] of PE time in moderate-to vigorous physical activity.

## Competing interests

The authors declare that they have no competing interests.

## Authors' contributions

JS conceived and designed the study. GS was responsible for recruitment, data collection, data entry, data analysis and drafting the manuscript. Both authors contributed to writing and approved the final manuscript.

## Pre-publication history

The pre-publication history for this paper can be accessed here:

http://www.biomedcentral.com/1471-2458/12/61/prepub
